# Supplementary material for: Acclimatization of a coral-dinoflagellate mutualism at a CO2 vent
Source: Commun Biol. 2023 Jan 18;6:66. doi: 10.1038/s42003-022-04327-3 (PMC9849335; doi:10.1038/s42003-022-04327-3)
Supplement: Supplementary file 2 — Supplementary Information [file 42003_2022_4327_MOESM2_ESM.pdf]

Acclimatization of a coral-algal mutualism at a CO_2 vent

Fiorella Prada[1,11,13,15], Silvia Franzellitti[2,11,15], Erik Caroselli[1,11]*, Itay Cohen[3], Mauro Marini[4,11], Alessandra Campanelli[4], Lorenzo Sana[2], Arianna Mancuso[1,11], Chiara Marchini[1,11], Alessia Puglisi[2], Marco, Candela[5,11], Tali Mass[6], Franco Tassi[7,12], Todd C. LaJeunesse[8]*, Zvy Dubinsky[9], Giuseppe Falini[10,11,14], Stefano Goffredo[1,11,14]*

SUPPLEMENTARY MATERIALS

Supplementary Figures

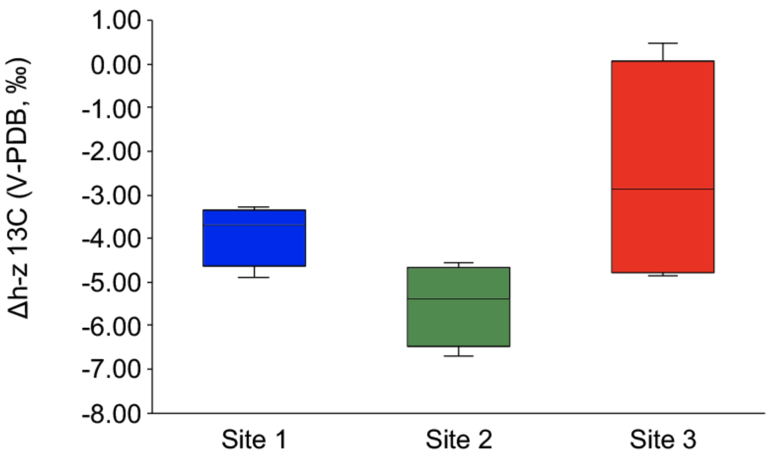

Supplementary Figure 1. Average $\Delta^{h\text{-}z}$ ^{13}C (V-PDB, ‰) values in *B. europaea* from Sites 1 (control), 2 (intermediate pCO_2) and 3 (high pCO_2). The boxes indicate the 25th and 75th percentiles and the line within the boxes mark the medians. Whisker length is equal to 1.5 × interquartile range (IQR).

SUPPLEMENTARY TABLES

Supplementary Table 1. Seawater carbonate chemistry data for each study site. Data are presented as mean values with 95% CIs in brackets. N = number of observations; T = temperature (number of observations); TA = total alkalinity; pCO_2 = carbon dioxide partial pressure; HCO_3^- = bicarbonate; CO_3^{2-} = carbonate; DIC = dissolved inorganic carbon; Ω_{arag} = aragonite saturation; NS = not significant; *p<0.05, ***p<0.001, Kruskal-Wallis equality-of- populations rank test. pH_{TS}, T, TA, and salinity were used to calculate the carbonate system parameters, denoted by (a). Pairwise comparisons between Sites were performed with Mann-Whitney U test. **p< 0.01, ***p< 0.001, NS = not significant

	**pH_{TS}**	**Salinity (‰)**	**T (°C)**	**TA (µmol/kg)**	**pCO_2 (µatm)[a]**	**HCO_3^- (µmol/kg)[a]**	**CO_3^{2-} (µmol/kg)[a]**	**CO_2 (µmol/kg)[a]**	**DIC (µmol/kg)[a]**	**Ω_{calc}[a]**	**Ω_{arag}[a]**
	7.97	35.6	21.1	2368	437	1888	207	13.7	2109	4.9	3.2
**Site 1**	(7.95-7.99)	(35.5-35.8)	(20.5-21.7)	(2277-2459)	(415-459)	(1867-1909)	(199-216)	(13.0-14.4)	(2094-2124)	(4.7-5.1)	(3.1-3.4)
	N = 198	N = 195	N = 198	N = 26	N = 195	N = 195	N = 195	N = 195	N = 195	N = 195	N = 195
	7.86	35.5	21.1	2288	654	1927	157	20.4	2105	3.7	2.5
**Site 2**	(7.83-7.90)	(35.3-35.7)	(20.5-21.8)	(2217-2359)	(589-720)	(1902-1952)	(150-165)	(18.5-22.3)	(2082-2127)	(3.3-3.9)	(2.3-2.6)
	N = 185	N = 185	N = 185	N = 25	N = 185	N = 185	N = 185	N = 185	N = 185	N = 185	N = 185
	7.64	35.6	21.1	2348	1161	1995	146	35.7	2177	3.5	2.3
**Site 3**	(7.55-7.77)	(35.4-35.8)	(20.5-21.7)	(2296-2399)	(832-1490)	(1973-2018)	(138-154)	(26.1-45.2)	(2155-2199)	(3.3-3.7)	(2.2-2.4)
	N = 189	N = 184	N = 189	N = 22	N = 184	N = 184	N = 184	N = 184	N = 184	N = 184	N = 184
**Pairwise comparisons**											
**1 vs 2**	***	NS	NS	NS	***	***	***	***	**NS**	***	***
**1 vs 3**	***	NS	NS	NS	***	***	***	***	**	***	***
**2 vs 3**	***	NS	NS	NS	***	***	***	***	**	**NS**	**NS**

Supplementary Table 2. Average concentration of sulphate (N = 5), phosphate (N = 4) and inorganic nitrogen (N = 4) in the seawater at the four sites. Data are presented as mean ± Standard Deviation (SD). The analytical error for sulphate is < 5%. Pairwise comparisons between Sites were performed with Mann-Whitney U test. *p< 0.05, **p< 0.01, NS = not significant

	**SO_4^{2-} (μmol L^{-1})**	**PO_4^{3-} (μmol L^{-1})**	**$NO_x(NO_2+NO_3)$ (μmol L^{-1})**
**Site 1**	36852 (±1243)	0.802 (± 1.355)	0.515 (± 0.450)
**Site 2**	35686 (± 2188)	0.158 (± 0.162)	0.113 (± 0.184)
**Site 3**	31855 (± 1001)	0.393 (± 0.419)	0.758 (± 0.997)
**Pairwise comparisons**			
**1 vs 2**	NS	NS	NS
**1 vs 3**	**	NS	NS
**2 vs 3**	*	NS	NS

Supplementary Table 3. Number of coral specimens measured by PAM fluorometry in the three Sites across the sampling period.

Date	Time interval	Number of corals			
		**Site 1**	**Site 2**	**Site 3**	**Total**
22/7/2019	18:00-20:00	10	10	10	30
23/7/2019	9:00-13:00	17	13	15	45
23/7/2019	20:00-22:00	11	8	11	30
24/7/2019	9:00-13:00	21	22	25	68
24/7/2019	20:00-22:00	14	9	15	38
25/7/2019	18:00-20:00	22	19	20	61
26/7/2019	18:00-20:00	19	19	19	57
14/2/2020	9:00-13:00	8	9	7	24
	**TOTAL**	**122**	**109**	**122**	**353**
	9:00-13:00	46	44	47	**137**
	18:00-20:00	51	48	49	**148**
	20:00-22:00	25	17	26	**68**

Supplementary Table 4. Average values for the three fluorescence parameters ΔF/Fm', F, and F’ at the three sites in the three time intervals.

		N	Mean (95% CI)
		**ΔF/Fm'**	
9:00-13:00	Site 1	46	0.52 (0.49-0.55)
	Site 2	44	0.53 (0.50-0.57)
	Site 3	47	0.55 (0.53-0.58)
		**F**	
	Site 1	46	258 (234-282)
	Site 2	44	303 (272-333)
	Site 3	47	314 (289-340)
		**Fm'**	
	Site 1	46	563 (503-623)
	Site 2	44	718 (604-832)
	Site 3	47	745 (658-832)
		**ΔF/Fm'**	
18:00-20:00	Site 1	51	0.60 (0.59-0.62)
	Site 2	48	0.61 (0.60-0.63)
	Site 3	49	0.59 (0.58-0.60)
		**F**	
	Site 1	51	351 (321-381)
	Site 2	48	443 (404-462)
	Site 3	49	421 (400-443)
		**Fm'**	
	Site 1	51	849 (783-916)
	Site 2	48	1113 (1049-1212)
	Site 3	49	1037 (984-1091)
		**ΔF/Fm'**	
20:00-22:00	Site 1	25	0.58 (0-56-0.61)
	Site 2	17	0.59 (0.54-0.64)
	Site 3	26	0.59 (0.57-0.62)
		**F**	
	Site 1	25	190 (172-208)
	Site 2	17	279 (248-310)
	Site 3	26	306 (288-324)
		**Fm'**	
	Site 1	25	482 (421-543)
	Site 2	17	740 (636-843)
	Site 3	26	769 (711-828)

Supplementary Table 5. Results of the PERMANOVA analysis for the three fluorescence parameters. Pairwise tests are shown for significant factors. df: degrees of freedom; SS: sum of squares; MS: mean squares. Significant differences are indicated in bold.

ΔF/Fm'

Source	df	SS	MS	Pseudo-F	P(perm)
Site	2	0.006	0.003	0.548	0.553
Hour	2	0.336	0.168	29.254	**0.001**
Site x Hour	4	0.026	0.006	1.115	0.357
Res	344	1.978	0.006		
Total	352	2.345			

Pairwise tests for the factor "Hour"

Groups	t	P(perm)
9:00-13:00 *vs* 18:00-20:00	7.323	**0.001**
9:00-13:00 *vs* 20:00-22:00	3.980	**0.001**
18:00-20:00 *vs* 20:00-22:00	1.416	0.155

F

Source	df	SS	MS	Pseudo-F	P(perm)
Site	2	0.424	0.212	28.588	**0.001**
Hour	2	1.298	0.649	87.590	**0.001**
Site x Hour	4	0.044	0.011	1.484	0.198
Res	344	2.550	0.007		
Total	352	4.3202			

Pairwise tests for the factor "Hour"

Groups	t	P(perm)
9:00-13:00 *vs* 18:00-20:00	10.008	**0.001**
9:00-13:00 *vs* 20:00-22:00	2.843	**0.008**
18:00-20:00 *vs* 20:00-22:00	11.537	**0.001**

Pairwise tests for the factor "Site"

Groups	t	P(perm)
Site 1 *vs* Site 2	5.497	**0.001**
Site 1 *vs* Site 3	7.388	**0.001**
Site 2 *vs* Site 3	0.743	0.460

Fm'

Source	df	SS	MS	Pseudo-F	P(perm)
Site	2	3.597	1.798	28.417	**0.001**
Hour	2	9.560	4.780	75.537	**0.001**
Site x Hour	4	0.343	0.086	1.356	0.232
Res	344	21.769	0.063		
Total	352	35.516			

Pairwise tests for the factor "Hour"

Groups	t	P(perm)
9:00-13:00 *vs* 18:00-20:00	10.392	**0.001**
9:00-13:00 *vs* 20:00-22:00	0.295	0.783
18:00-20:00 *vs* 20:00-22:00	10.653	**0.001**

Pairwise tests for the factor "Site"

Groups	t	P(perm)
Site 1 *vs* Site 2	6.16	**0.001**
Site 1 *vs* Site 3	7.4738	**0.001**
Site 2 *vs* Site 3	0.31678	0.769

Supplementary Table 6. δ^{13}C, δ^{15}N and C/N ratios for host tissue and algal symbionts in Sites 1 (control), 2 (intermediate pCO_2) and 3 (high pCO_2). Pairwise comparisons between Sites were performed with a post-hoc Fisher least significant differences test (LSD). N = 4 specimens for each Site. NS: Not Significant, **p<0.01, ***p< 0.001.

				Pairwise comparison		
		Tissue	Algal symbiont	Sites	Tissue	Algal symbiont
$\delta^{13}C$ (‰)	Site 1	-19.55	-16.27	1 vs 2	NS	NS
		-19.99	-15.09			
		-19.20	-15.64			
		-18.08	-14.26			
	Site 2	-18.89	-13.95	1 vs 3	NS	NS
		-19.13	-14.57			
		-21.41	-15.59			
		-19.68	-12.98			
	Site 3	-17.95	-13.43	2 vs 3	NS	NS
		-17.93	-18.40			
		-19.41	-18.21			
		-19.55	-14.67			
$\delta^{15}N$ (‰)	Site 1	3.57	3.10	1 vs 2	***	NS
		3.05	2.31			
		3.92	1.69			
		3.86	1.53			
	Site 2	0.80	1.50	1 vs 3	***	NS
		-0.64	1.82			
		-0.71	1.92			
		0.03	0.65			
	Site 3	0.93	0.56	2 vs 3	NS	NS
		0.83	0.49			
		-0.04	0.74			
		0.03	3.26			
C/N ratio	Site 1	5.60	6.56	1 vs 2	***	**
		4.95	6.36			
		5.76	6.40			
		6.16	7.45			
	Site 2	1.14	8.67	1 vs 3	***	***
		0.32	8.10			
		0.09	7.56			
		0.19	9.78			
	Site 3	1.26	11.25	2 vs 3	NS	**
		1.94	11.00			
		0.24	10.72			
		0.46	9.86			
