# Supplementary material for: Acclimatization of a coral-dinoflagellate mutualism at a CO2 vent
Source: Commun Biol. 2023 Jan 18;6:66. doi: 10.1038/s42003-022-04327-3 (PMC9849335; doi:10.1038/s42003-022-04327-3)
Supplement: Supplementary file 3 — Description of Additional Supplementary Files [file 42003_2022_4327_MOESM3_ESM.pdf]

Description of Additional Supplementary Files

File name: Supplementary Data 1
Description: Alignment of psbA non-coding region haplotype sequences for *Philozoon balanophyllum.*

File name: Supplementary Data 2
Description: The source data behind the graphs in the paper.
